# Epidemiological profile of diabetics who died in Brazil from 2008 to 2012

**DOI:** 10.1186/1758-5996-7-S1-A191

**Published:** 2015-11-11

**Authors:** Erika Cesar de Oliveira Naliato, Vctor Luiz Sepúlveda Rey, Ramon Gabriel Souza de Oliveira

**Affiliations:** 1 Centro de Estudos Ricardo A T Castilho-Associação Médica De Teresópolis, Teresópolis, Brazil

## Background

Brazil has a high prevalence of Diabetes Mellitus (DM) and this disease is known to responsible for an increase in mortality.

## Objective

To determine the epidemiological profile of patients with DM who died in Brazil in the period correspondent to 2008-2012, using information available at the DATASUS data bank.

## Materials and methods

The Mortality Information System of the DATASUS data bank (Ministry of Health-Brazil) was accessed in order to allow the analysis of the following variables related to subjects with DM: age, gender, race, marital status, degree of education, type of DM, place of demise, and country region and Federation state where the casualty was reported.

## Results

According to the analysis of the DATASUS databank, 30,163 death certificates from 2008 to 2012 revealed that the deceased had the diagnosis of DM. More than half of them were women (56.33%) and Caucasians (53.58%). Regarding their marital status, more than a third of them were married (39.65%). Most deaths happened in type 2 diabetics (66.82%), achieving a proportion of 2.01 deaths of patients with type 2 for each one with type 1 DM. Most deaths occurred during a hospitalization period (65.29%), but a significant percentage of patients died at home (27.52%). In addition, fewer casualties happened in other Health institutions (4.04%), other settings (2.12%), and even on the streets (0.89%), while in 0.14% the place of demise was ignored. The deaths concentrated in the older age groups: 77.83% occurred in patients aged 60 yrs. or older (60-69 yrs.=22.12%; 70-79 yrs.=27.81%; 80 yrs. and above=27.9%). The majority of the casualties involved patients with none (22.19%) or few yrs. of formal education (1-3 yrs.=24.87%; 4-7 yrs.=17.82%). The Southeast region of Brazil responded for the greatest number of deaths (33.78%), followed by the Northeast (28.81%) and the South (22.78%), with fewer cases in the North (8.43%) and West Central (6.19%) regions. Among the Federation states, São Paulo had the highest number of deaths (4,702), followed by Paraná (2,807), Rio Grande do Sul (2,763), and Rio de Janeiro (2,548). The epidemiological data of the deaths in types 1 and 2 diabetes were similar.

## Conclusion

Casualties in Brazilian diabetics concentrated in the Southeast region of the country and in hospitalization settings, involving especially type 2 patients aged 60 yrs. or above with a lower educational profile.

